# Compromised Blood–Brain Barrier Integrity Is Associated With Total Magnetic Resonance Imaging Burden of Cerebral Small Vessel Disease

**DOI:** 10.3389/fneur.2018.00221

**Published:** 2018-04-06

**Authors:** Yue Li, Man Li, Long Zuo, Qinglei Shi, Wei Qin, Lei Yang, Tao Jiang, Wenli Hu

**Affiliations:** ^1^Department of Neurology, Beijing Chao-Yang Hospital, Capital Medical University, Beijing, China; ^2^Department of Radiology, Beijing Chao-Yang Hospital, Capital Medical University, Beijing, China; ^3^Diagnosis Imaging, Siemens Healthcare Ltd., Beijing, China

**Keywords:** cerebral small vessel disease, blood–brain barrier, lacunes, white matter hyperintensities, cerebral microbleeds, enlarged perivascular spaces, dynamic contrast-enhanced-magnetic resonance imaging

## Abstract

**Objective:**

Several studies have demonstrated that compromised blood–brain barrier (BBB) integrity may play a pivotal role in the pathogenesis of individual cerebral small vessel disease (cSVD) markers, but the association between BBB permeability and total magnetic resonance imaging (MRI) cSVD burden remains unclear. This study aimed to investigate the relationship between BBB permeability and total MRI cSVD burden.

**Methods:**

Consecutive participants without symptomatic stroke history presented for physical examination were enrolled in this cross-sectional study. The presence of lacunes, white matter hyperintensities (WMH), cerebral microbleeds, and enlarged perivascular spaces was recorded in an ordinal score (range 0–4). We used dynamic contrast-enhanced-MRI and Patlak pharmacokinetic model to quantify BBB permeability in the normal-appearing white matter (NAWM), WMH, cortical gray matter (CGM), and deep gray matter (DGM).

**Results:**

All 99 participants averaged 70.33 years old (49–90 years). Multivariable linear regression analyses adjusted for age, sex, and vascular risk factors showed that leakage rate and area under the leakage curve in the NAWM, WMH, CGM, and DGM were positively associated with total MRI cSVD burden (all *P* < 0.01). Moreover, fractional blood plasma volumes in the NAWM, CGM, and DGM were negatively associated with total MRI cSVD burden (all *P* < 0.05).

**Conclusion:**

This study verified that compromised BBB integrity is associated with total MRI cSVD burden, suggesting that BBB dysfunction may be a critical contributor to the pathogenesis of cSVD. Longitudinal studies are required to determine whether there is a causal relationship between BBB permeability and total MRI cSVD burden.

## Introduction

Cerebral small vessel disease (cSVD) is a general term commonly used to describe a group of pathological processes involving perforating cerebral arterioles, capillaries, and venules (1). Lacunes, white matter hyperintensities (WMH), cerebral microbleeds (CMBs), and enlarged perivascular spaces (EPVS) have been identified as magnetic resonance imaging (MRI) markers of cSVD (2). CSVD is associated with an increased risk of stroke, cognitive impairment, and gait abnormalities (3). The pathogenesis of cSVD has not been completely understood but evidence is amounting that blood–brain barrier (BBB) dysfunction is a contributing factor (4).

Dynamic contrast-enhanced (DCE)-MRI combined with an appropriate pharmacokinetics model is a reliable method to quantitatively evaluate BBB permeability (5). Using DCE-MRI method, previous cross-sectional studies reported compromised BBB integrity in patients with lacunar stroke, WMH, and mild vascular cognitive impairment (mVCI) (6–10). Moreover, a longitudinal study (11) revealed the association between poor functional outcome and increased BBB permeability in cSVD patients. However, these studies mostly focused on single MRI markers of cSVD, and no studies have investigated the combined effects of cSVD features to date.

Recently, Staals et al. (12) proposed a validated scale to evaluate comprehensive cSVD burden (range 0–4) by summing different MRI features, including lacunes, WMH, CMBs, and EPVS. Since the four cSVD markers are often correlated instead of occurring separately, this total MRI cSVD burden may be a more appropriate method to represent their combined effects.

A previous study (8) has chosen the normal-appearing white matter (NAWM), WMH, cortical gray matter (CGM), and deep gray matter (DGM) as regions of interest (ROIs) and demonstrated a larger tissue volume with subtle BBB leakage in cSVD patients than in the controls, thus supporting the generalized nature of cSVD. This study aimed to verify whether BBB permeability increased with the aggravation of total MRI cSVD burden in these ROIs. To exclude the effect of symptomatic stroke on BBB permeability (13), participants with symptomatic stroke history were not selected for this study. In addition, we tentatively examined whether each of the MRI markers was independently associated with BBB permeability change.

## Materials and Methods

### Study Population

We recruited consecutive participants presented for physical examination at the department of Neurology in Beijing Chao-Yang Hospital, Capital Medical University, from May 2016 to April 2017. Exclusion criteria included: (1) history of symptomatic stroke or carotid stenosis of ≥50%, epilepsy, Alzheimer disease, neurodegenerative disease, and other neurological disorders; (2) tumor, brain trauma, systemic inflammatory disease; (3) contraindication for MRI (e.g., metal implants, pacemaker, and claustrophobia) or the use of the contrast agent (e.g., renal failure or allergy to gadolinium); and (4) alcohol or drug abuse, psychiatric disorders (e.g., depression or schizophrenia).

### Ethics Statement

All participants consented to participate in our study and signed an informed consent to the use of data for research. The design of this study was approved by the Ethics Committee of Beijing Chao-Yang Hospital, Capital Medical University and was performed in accordance with the declaration of Helsinki.

### MRI Protocol and Assessment

#### Structural MRI

All participants underwent structural brain MRI on a 3T MRI scanner (Prisma; Siemens AG, Erlangen, Germany). Sequences included diffusion-weighted imaging, T1-weighted (T1-W), T2-weighted (T2-W), fluid-attenuated inversion recovery (FLAIR), and susceptibility-weighted imaging (SWI), respectively. MRI sequence parameters are provided in Table S1 in Supplementary Material.

#### Dynamic Contrast-Enhanced (DCE)-MRI

Magnetic resonance imaging examinations were performed on a 3T MRI scanner (Prisma; Siemens AG, Erlangen, Germany). T1 dynamic protocol comprises precontrast T1 measurements with two different flip angles (3°, 15°) for T1 mapping, as well as continuous serial acquisitions of 60 volumes of T1-W images. The sequence was applied [repetition time (TR)/echo time (TE) 5.08/1.8 ms, field of view 230 mm × 230 mm, voxel size 1.2 mm × 1.2 mm × 3 mm]. After start of acquisition of four volumes of T1-W images, the contrast agent (gadolinium, 1.0 mmol/mL; 0.1 mmol/kg body weight, range 5–10 mmol per person) was administered in the antecubital vein at a rate of 2.5 mL/s using a power injector, followed by a 20 mL saline flush.

#### MR Imaging Analysis

DCE-MRI data were processed offline using Nordic ICE (Nordic Neuro Lab, Bergen, Norway). The concentration of contrast agent in tissue was calculated using relative signal change and T1 mapping. Individual vascular input functions were derived from the superior sagittal sinus (14) using a semi-automated method in the Nordic ICE (Nordic Neuro Lab) software. The Patlak graphical approach that was identified as the most appropriate model for low-leakage regimen was applied per voxel (15). The Patlak graphical approach provided BBB leakage rate (*K*_trans_), area under the leakage curve (AUC), and fractional blood plasma volume (*V*_p_).

#### Regions of Interest

**Figure 1:**
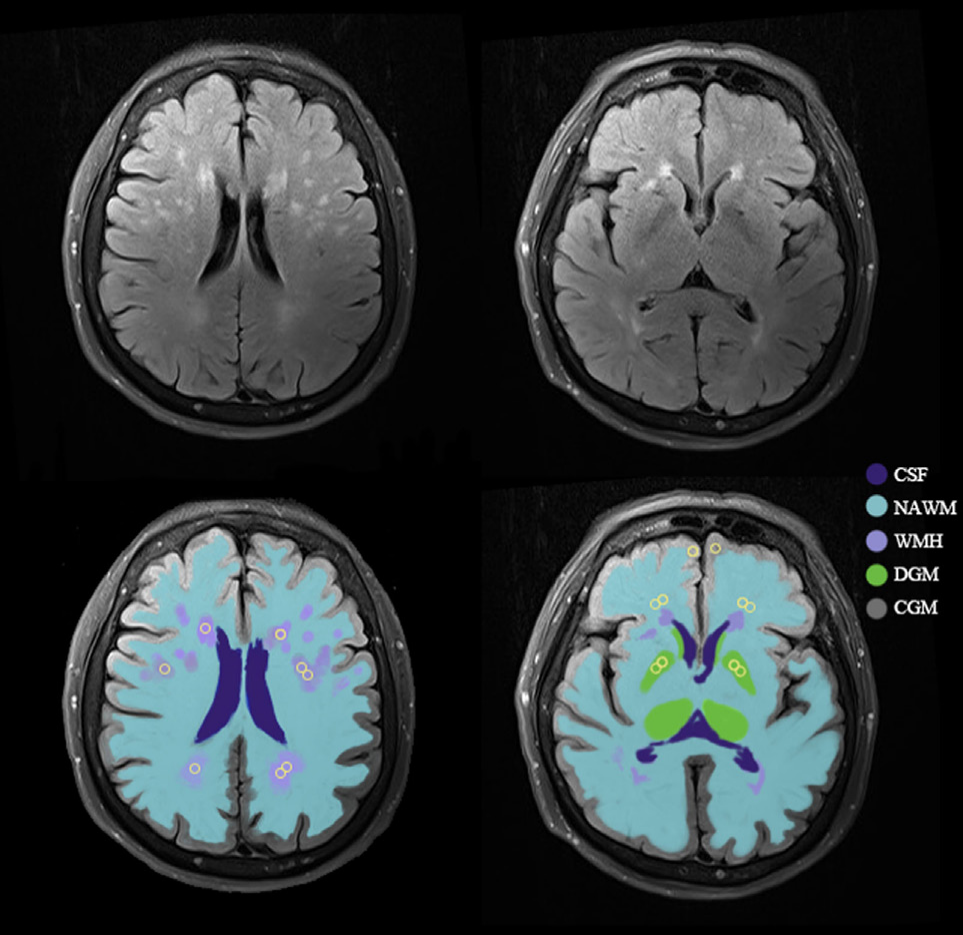
An example of tissue segmentation and regions of interest (ROIs). Example of the template for sampling ROIs (yellow circles) in normal-appearing white matter (NAWM), white matter hyperintensities (WMH), cortical gray matter (CGM), and deep gray matter (DGM). Abbreviation: CSF, cerebrospinal fluid.

We used an axial T2 FLAIR MRI sequence with the same orientation and slice thickness as our DCE sequence to manually draw ROIs in the NAWM, WMH, CGM, and DGM in both hemispheres (Figure 1) (16). CGM was placed at prefrontal cortex (size = 5 mm^2^) and DGM was placed at lentiform nucleus (size = 5 mm^2^). Since WMH were divided into periventricular WMH (PVWMH) and deep WMH (DWMH), we drew ROIs in both areas that were hyperintensive (size = 5 mm^2^). NAWM (size = 5 mm^2^) was placed around the WMH lesions. For participants without visible WMH, we strived to match the anatomical location and size of the ROIs as close as possible. The WMH was placed around the periventricular, and NAWM was located in the 10 mm area around the WMH area. Each ROI was measured for four times and averaged to obtain the average BBB leakage parameters. An experienced radiologist performed this procedure manually.

#### Assessment of Total MRI cSVD Burden

Neuroimaging markers of cSVD were defined according to Standards for Reporting Vascular Changes on Neuroimaging (STRIVE) criteria (2). Lacunes were defined as round or ovoid fluid-filled cavities of 3–15 mm on T2-W and FLAIR (2). DWMH and PVWMH were graded using the Fazekas scale (17). CMBs were defined as round or ovoid lesions of ≤10 mm with low signal intensity on SWI and categorized according to Microbleed Anatomical Rating Scale (18). EPVS were identified as punctate or linear hyperintensities on T2-W images in the basal ganglia (BG) or centrum semiovale. A 4-point visual rating ordinal scale (0, no EPVS; 1, ≤10; 2, 11–20 EPVS; 3, 21–40 EPVS; 4, >40 EPVS) was used to evaluate the severity of EPVS (19).

We used the recently reported scale to represent the total MRI cSVD burden by counting the presence of each of the four features of cSVD (12). A point was awarded for each of the following items: ≥1 lacune; Fazekas score ≥2 in deep white matter (DWM) and/or Fazekas score of 3 in periventricular white matter (PVWM); ≥1 deep or infratentorial CMBs (20); moderate to extensive (grade 2–4) EPVS in the BG (19). Hence, the score ranged from 0 to 4 points.

All images were analyzed by two experienced radiologists blinded to the clinical data. An interobserver reliability test was performed in 35 subjects, and the κ-coefficient for lacunes, WMH, CMBs, and EPVS was 0.815, 0.792, 0.832, and 0.791, respectively. Disagreement was resolved by discussing with other coauthors.

### Statistical Analysis

Continuous variables with normal distribution were presented as mean with SD and compared using one-way analysis of variance, followed by Student–Newman–Kuels multiple comparison test. Variables with non-normal distribution were presented as median with interquartile ranges and compared using Kruskal–Wallis test. Bonferroni correction was used for *post hoc* comparisons. Categorical variables were compared using chi-square tests. The associations between BBB permeability parameters and total MRI cSVD burden were examined using Spearman correlation analysis. Subsequently, the association between BBB permeability with total MRI cSVD burden was investigated using univariable linear regression analyses. Multivariable linear regression analyses were then used to adjust for age, sex, and vascular risk factors.

Coefficients of determination (*R*^2^) were calculated in univariable linear regression analyses to determine the proportion of variance in BBB permeability explained by total MRI cSVD burden. To investigate the contribution of each of the MRI markers, we repeated the analysis with lacunes, WMH, CMBs, and EPVS as independent variables individually (dichotomized, as defined above) and *R*^2^ were calculated. In addition, *R*^2^ of Fazekas score (range 0–6) were calculated. Statistical significance was established at *P* < 0.05. Analysis was performed with Statistical Package for Social Sciences (version 24).

## Results

### Participants Characteristics

A total of 139 participants were recruited but 40 were excluded (12 participants with incomplete injection of contrast or contraindications for MRI, 18 participants with history of symptomatic stroke or carotid stenosis, and the other 10 participants with history of tumor). In the end, 99 participants (70.33 ± 9.07 years; 49.5% male) were enrolled.

For total MRI cSVD burden, 31 (31.31%) participants had a total cSVD score of 0; 25 (25.25%), 1; 16 (16.16%), 2; 15 (15.15%), 3; and 12 (12.12%), 4, respectively. The prevalence of each cSVD marker was lacunes, 38 (38.38%); WMH, 47 (47.47%); CMBs, 24 (24.24%); and EPVS, 41 (41.41%), respectively. Clinical characteristics of participants are presented in Table 1. There was no significant difference in baseline characteristics and laboratory tests (Table S2 in Supplementary Material) among five groups.

**Table 1:** Demographic and clinical features of participants with different severity of total MRI cSVD burden.

	Total (*n* = 99)	cSVD 0 (*n* = 31)	cSVD 1 (*n* = 25)	cSVD 2 (*n* = 16)	cSVD 3 (*n* = 15)	cSVD 4 (*n* = 12)	*P*
Male, *n* (%)	49 (49.5)	13 (41.9)	15 (60.0)	5 (31.3)	8 (53.3)	8 (66.7)	0.244
Age, years	70.33 ± 9.07	67.42 ± 9.04	70.44 ± 7.88	71.06 ± 7.86	75.47 ± 10.22	70.25 ± 9.87	0.722
Hypertension, *n* (%)	65 (65.7)	16 (51.6)	17 (68.0)	11 (68.8)	13 (86.7)	8 (66.7)	0.188
Diabetes mellitus, *n* (%)	19 (19.2)	5 (16.1)	2 (8.0)	4 (25.0)	3 (20.0)	5 (41.7)	0.186
Hyperlipidemia, *n* (%)	53 (52.5)	14 (45.2)	14 (56.0)	9 (56.3)	9 (60.0)	6 (50.0)	0.870
Current smoking, *n* (%)	27 (27.3)	6 (19.4)	9 (36.0)	5 (31.3)	3 (20.0)	4 (33.3)	0.605
BMI, kg/m^2^	25.79 ± 3.14	25.51 ± 2.49	26.37 ± 4.17	26.07 ± 2.66	25.37 ± 3.40	25.44 ± 2.66	0.243

*Data are presented as mean ± SD, median (interquartile range), or counts (%)*.

*MRI, magnetic resonance imaging; cSVD, cerebral small vessel disease; n, number of persons; BMI, body mass index*.

### Association Between BBB Permeability and Total MRI cSVD Burden

**Figure 2:**
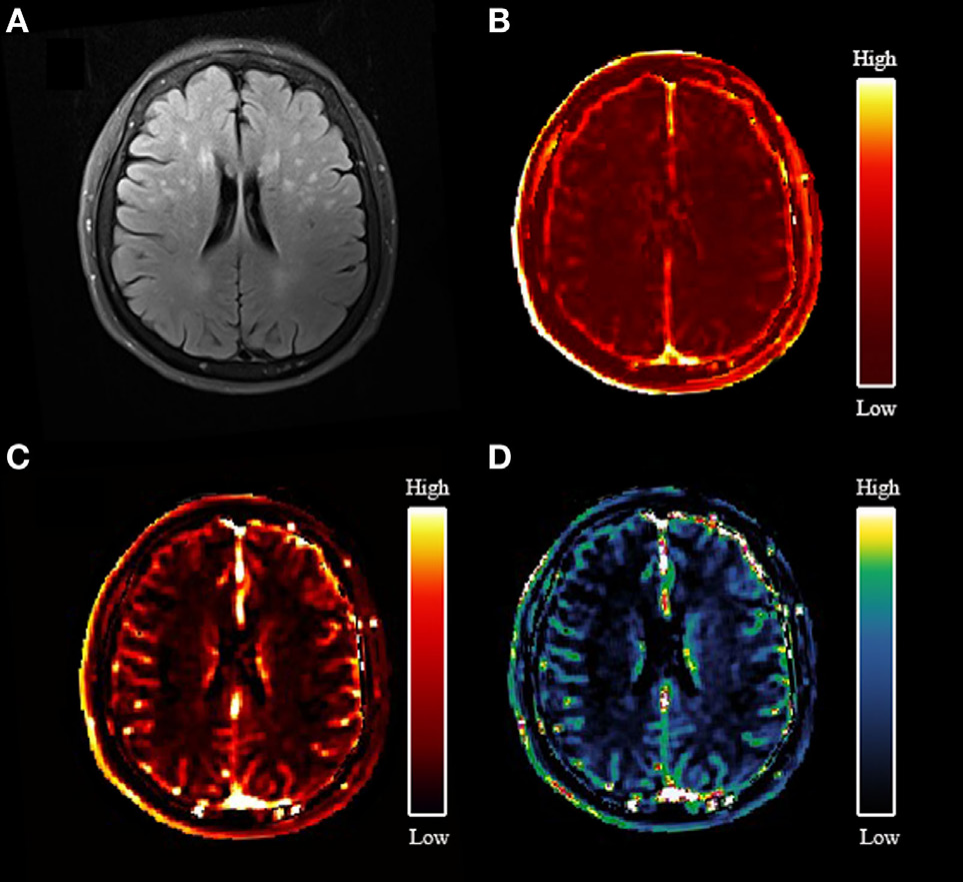
An example map. **(A)** Axial fluid-attenuated inversion recovery image of a 77-year-old woman; **(B)** blood–brain barrier leakage rate (*K*_trans_) map; **(C)** area under the leakage curve map; **(D)** fractional blood plasma volume (*V*_p_) map.

An example of leakage rate, area under the leakage curve, and fractional blood plasma volume map is displayed in Figure 2. An overview of the quantitative results and statistical results is shown in Table 2. Spearman correlation analysis revealed that in all ROIs, BBB leakage rate, and area under the leakage curve were both positively correlated with total MRI cSVD burden, while blood plasma volume in the NAWM, CGM, and DGM showed negative correlation with total MRI cSVD burden.

**Table 2:** Leakage rate, area under the leakage curve, and fractional blood plasma volume of participants with different severity of total MRI cSVD burden.

	cSVD 0 (*n* = 31)	cSVD 1 (*n* = 25)	cSVD 2 (*n* = 16)	cSVD 3 (*n* = 15)	cSVD 4 (*n* = 12)	*P*
**NAWM**						
*K*_trans_ (10^−4^ min^−1^)	0.14 (0.05, 0.32)^a^^,^^b^^,^^c^	0.18 (0.11, 0.36)^e^^,^^f^	0.30 (0.22, 0.43)^a^	0.37 (0.22, 0.48)^b^^,^^e^	0.47 (0.35, 0.55)^c^^,^^f^	<0.001
AUC	3.31 ± 1.14^a^^,^^b^^,^^c^	3.57 ± 0.99^d^^,^^e^^,^^f^	4.75 ± 0.64^a^^,^^d^	4.45 ± 1.43^b^^,^^e^	4.93 ± 1.43^c^^,^^f^	<0.001
*V*_p_ (10^−2^)	6.85 ± 2.64^a^^,^^b^	5.99 ± 2.45	4.76 ± 2.21^a^	4.73 ± 2.00^b^	5.29 ± 1.90	0.015
**WMH**						
*K*_trans_ (10^−4^ min^−1^)	0.28 (0.11, 0.43)^a^^,^^b^^,^^c^	0.36 (0.19, 0.54)^f^	0.57 (0.33, 0.94)^a^	0.56 (0.39, 0.87)^b^	0.67 (0.55, 0.93)^c,f^	<0.001
AUC	4.00 ± 1.34^a^^,^^b^^,^^c^	4.41 ± 1.31^d^^,^^e^^,^^f^	6.20 ± 1.89^a^^,^^d^	6.50 ± 1.79^b^^,^^e^	7.05 ± 1.82^c^^,^^f^	<0.001
*V*_p_ (10^−2^)	9.49 ± 5.04	8.70 ± 4.70	9.37 ± 5.15	8.43 ± 5.28	6.62 ± 3.10	0.497
**CGM**						
*K*_trans_ (10^−4^ min^−1^)	0.93 (0.70, 1.26)^a^^,^^b^^,^^c^	0.97 (0.77, 1.29)^e^^,^^f^	1.36 (0.99, 2.39)^a^	1.91 (1.12, 2.44)^b^^,^^e^	1.59 (1.21, 2.03)^c^^,^^f^	<0.001
AUC	14.85 ± 5.96^a^^,^^b^^,^^c^	15.64 ± 4.89^d^^,^^f^	19.22 ± 4.81^a^^,^^d^	18.55 ± 4.98^b^	20.02 ± 5.29^c^^,^^f^	0.008
*V*_p_ (10^−2^)	27.66 ± 9.91^c^	24.65 ± 9.25^f^	23.42 ± 9.84	22.20 ± 10.05	17.91 ± 7.04^c^^,^^f^	0.043
**DGM**						
*K*_trans_ (10^−4^ min^−1^)	0.51 (0.30, 0.74)^b^^,^^c^	0.61 (0.44, 0.90)^f^	0.72 (0.58, 1.05)	0.96 (0.67, 1.53)^b^	1.00 (0.91, 1.47)^c^^,^^f^	<0.001
AUC	9.54 ± 3.24^a^^,^^c^	10.51 ± 2.26	11.92 ± 2.15^a^	11.17 ± 2.88	12.37 ± 2.06^c^	0.009
*V*_p_ (10^−2^)	18.37 ± 6.41^c^	18.30 ± 8.06^f^	14.75 ± 5.86	14.14 ± 6.19	11.56 ± 5.44^c^^,^^f^	0.011

*Data are presented as mean ± SD or median (interquartile range)*.

*MRI, magnetic resonance imaging; cSVD, cerebral small vessel disease; NAWM, normal-appearing white matter; WMH, white matter hyperintensities; CGM, cortex gray matter; DGM, deep gray matter; K_trans_, leakage rate; AUC, area under the leakage curve; V_p_, fractional blood plasma volume*.

*^a^Significant difference between cSVD 0 and cSVD 2 categories*.

*^b^Significant difference between cSVD 0 and cSVD 3 categories*.

*^c^Significant difference between cSVD 0 and cSVD 4 categories*.

*^d^Significant difference between cSVD 1 and cSVD 2 categories*.

*^e^Significant difference between cSVD 1 and cSVD 3 categories*.

*^f^Significant difference between cSVD 1 and cSVD 4 categories*.

Univariable linear regression analysis revealed that leakage rate and area under the leakage curve in all ROIs were positively associated with total MRI cSVD burden while fractional blood plasma volume in the NAWM, CGM, and DGM was negatively associated with total MRI cSVD burden. These associations remained significant after adjustment for age, sex, and vascular risk factors. These statistical results are displayed in Table 3.

**Table 3:** Association of leakage rate, area under the leakage curve, and fractional blood plasma volume with total MRI cSVD burden.

	Spearman correlation		Univariable^a^		Multivariable^b^	
	*r*	*P* value	β	*P* value	β	*P* value
**NAWM**						
*K*_trans_	0.558	<0.001	0.082	<0.001	0.082	<0.001
AUC	0.444	<0.001	0.432	<0.001	0.432	<0.001
*V*_p_	−0.275	0.006	−0.523	0.003	−0.523	0.003
**WMH**						
*K*_trans_	0.573	<0.001	0.149	<0.001	0.154	<0.001
AUC	0.583	<0.001	0.839	<0.001	0.867	<0.001
*V*_p_	−0.135	0.183	−0.528	0.348	−0.133	0.184
**CGM**						
*K*_trans_	0.500	<0.001	0.245	<0.001	0.230	<0.001
AUC	0.378	<0.001	1.379	0.001	1.608	<0.001
*V*_p_	−0.282	0.005	−2.155	0.002	−2.082	0.033
**DGM**						
*K*_trans_	0.489	<0.001	0.173	<0.001	0.173	<0.001
AUC	0.351	<0.001	0.672	0.001	0.602	0.003
*V*_p_	−0.323	0.001	−1.720	0.001	−1.623	0.001

*MRI, magnetic resonance imaging; cSVD, cerebral small vessel disease; NAWM, normal-appearing white matter; WMH, white matter hyperintensities; CGM, cortex gray matter; DGM, deep gray matter; K_trans_, leakage rate; AUC, area under the leakage curve; V_p_, fractional blood plasma volume*.

*^a^Univariable linear regression analysis with K_trans_, AUC, and V_p_, respectively, as dependent variable, and total MRI cSVD burden as independent variable*.

*^b^Multivariable linear regression analysis with K_trans_, AUC, and V_p_ as dependent variable and age, sex, total MRI cSVD burden, and vascular risk factors as independent variables*.

*β, Unstandardized regression coefficient*.

In our research, there are five participants who did not have visible WMH, their WMH ROI was placed around the periventricular, which may cause an ambiguity. Thus, we tentatively took out these five participants and repeated the statistical analysis. Leakage rate and area under the leakage curve in the NAWM, WMH, CGM, and DGM were still positively correlated with total MRI cSVD burden; and the association between higher total MRI cSVD burden and lower fractional blood plasma volume in the NAWM, CGM, and DGM still held. These statistical results are displayed in Tables S3–S5 in Supplementary Material.

### Correlations of Determination of Individual MRI Markers

*R*^2^, which indicated the proportion of variance in BBB permeability explained by total MRI cSVD burden and each of individual MRI markers, are displayed in Table 4. The magnitude of the individual MRI markers differed across parameters. The dichotomized WMH explained a higher proportion of variance in leakage rate and area under the leakage curve than the presence of lacunes and CMBs in most of ROIs. The proportions of variance in BBB leakage explained by Fazekas score were comparable to those explained by total MRI cSVD burden. Fazekas score explained a higher proportion of variance in fractional blood plasma volume in NAWM, WMH, and CGM than total MRI cSVD burden.

**Table 4:** *R*^2^ for the association between BBB permeability and total MRI cSVD burden versus individual MRI markers.

	Total MRI cSVD burden		Lacunes		WMH		Fazekas score		CMBs		EPVS	
	*R*^2^	*P*	*R*^2^	*P*	*R*^2^	*P*	*R*^2^	*P*	*R*^2^	*P*	*R*^2^	*P*
**NAWM**												
*K*_trans_	0.220	<0.001	0.150	<0.001	0.127	<0.001	0.161	<0.001	0.106	0.001	0.082	0.004
AUC	0.170	<0.001	0.054	0.021	0.194	<0.001	0.263	<0.001	0.133	<0.001	0.049	0.028
*V*_p_	0.087	0.003	0.058	0.017	0.054	0.021	0.170	<0.001	0.008	0.392	0.077	0.005
**WMH**												
*K*_trans_	0.265	<0.001	0.079	0.005	0.164	<0.001	0.243	<0.001	0.113	0.001	0.214	<0.001
AUC	0.357	<0.001	0.114	0.001	0.353	<0.001	0.364	<0.001	0.207	<0.001	0.118	0.001
*V*_p_	0.023	0.132	0.042	0.041	0.003	0.601	0.048	0.030	0.005	0.479	0.012	0.288
**CGM**												
*K*_trans_	0.181	<0.001	0.141	<0.001	0.108	0.001	0.167	<0.001	0.036	0.060	0.105	0.001
AUC	0.117	0.001	0.018	0.190	0.123	<0.001	0.086	0.003	0.054	0.020	0.067	0.010
*V*_p_	0.094	0.002	0.092	0.002	0.039	0.051	0.124	<0.001	0.037	0.055	0.037	0.058
**DGM**												
*K*_trans_	0.233	<0.001	0.128	<0.001	0.146	<0.001	0.216	<0.001	0.115	0.001	0.101	0.001
AUC	0.110	0.001	0.075	0.006	0.094	0.002	0.100	0.001	0.034	0.066	0.035	0.064
*V*_p_	0.117	0.001	0.084	0.004	0.034	0.069	0.106	0.001	0.011	0.291	0.039	0.051

*R^2^, coefficient of determination; cSVD, cerebral small vessel disease; WMH, white matter hyperintensities; CMBs, cerebral microbleeds; EPVS, enlarged perivascular spaces; NAWM, normal-appearing white matter; CGM, cortex gray matter; DGM, deep gray matter; K_trans_, leakage rate; AUC, area under the leakage curve; V_p_, fractional blood plasma volume; MRI, magnetic resonance imaging; BBB, blood–brain barrier*.

Since the results showed that Fazekas score explained similar or even higher proportions of variance in BBB permeability than the total score, we tentatively examined whether the association still existed if WMH was taken out from the total score. Thus, we designed a new “total score” scale in which WMH was taken out and repeated the statistical analysis. Leakage rate and area under the leakage curve in the NAWM, WMH, CGM, and DGM were still positively correlated with total MRI cSVD burden; and the relationship between higher total MRI cSVD burden and lower fractional blood plasma volume in the NAWM, CGM, and DGM still held. We also calculated the *R*^2^ of this new “total score”. These statistical results are displayed in Tables S6 and S7 in Supplementary Material. These results showed that other features of cSVD except for WMH also associated with BBB permeability.

## Discussion

The main finding of this study was that BBB permeability in the NAWM, WMH, CGM, and DGM measured by DCE-MRI was positively correlated with total MRI cSVD burden. We also found an association between higher total MRI cSVD burden and lower fractional blood plasma volume in the NAWM, CGM, and DGM.

Previous studies paid much attention to the individual MRI features of cSVD and described associations between compromised BBB integrity and lacunar stroke or WMH (6, 8). We think it is necessary to evaluate the association between total MRI cSVD burden and BBB permeability since total MRI cSVD burden could represent the underlying severity of cSVD better than single MRI markers. Our study comprehensively clarified the association of BBB permeability and cerebral blood flow (CBF) with total MRI cSVD burden.

Although lacunes and WMH are often found together, the independent relationship between WMH and BBB leakage has been frequently reported. Using diffusion tensor imaging and DCE-MRI in combination, Munoz Maniega et al. (9) found that BBB permeability increased with the rise of WMH burden, but they only described BBB leakage rate of WMH and NAWM, and did not evaluate those in the cortex. Zhang et al. (8) found the leakage volume of the NAWM, WMH, and CGM in cSVD and mVCI patients was higher compared with controls, but there was no significant difference in leakage rate in all ROIs. The discrepancy between this study and our study might be explained by differences in the study participants. We excluded participants with a history of symptomatic stroke since stroke may also cause BBB permeability change (13).

The precise mechanism for the relationship between BBB permeability and cSVD is not well established. An experiment was carried out with spontaneously hypertensive stroke-prone rats (SHRSP), and it was conjectured that BBB breakdown may be the starting point of cSVD (21). The change of BBB permeability might mediate a series of pathophysiological changes and eventually lead to cSVD. BBB is a selective barrier structure consists of capillary endothelial cells, pericytes, astrocytes and extracellular matrix (22). Tight junctions (TJs) are an important component of BBB which couple endothelial cells mechanically and prevent toxic substances from leaking into the brain interstitium (23). The breakdown of TJs will result in leakage of plasma content, change of cell polarity, and disorder of transport mechanism (24). Extravasation of intravascular substances will cause hyaline degeneration of small vessel walls, perivascular edema, and neuronal toxicity damage, and then lead to decreased nerve fiber density, myelinoclasis, oligodendrocyte axon damage, astrocyte proliferation, swelling, neurite collapse, and microglial cell activation (25). These series of events could explain the pathological and imaging features of lacunar infarctions, WMH, CMBs, and EPVS (4). There is a new insight that barrier changes in cSVD is at the capillary level, which is typically characterized by massive losses of smooth muscle cells and some arterioles could contribute to the loss of cerebrovascular barrier protection (26).

Notably, the association between CMBs and BBB permeability indicated that BBB dysfunction may be a contributor to the pathogenesis of CMBs. A previous study (27) showed that CMBs were linked to the deposition of β-amyloid in small vessel walls while another study (28) revealed that iron deposition might be an indicator of cSVD that predisposes to WMH. These depositions may be attributed to the altered transport systems and deteriorated environment of the neural cells caused by compromised BBB integrity (22).

A number of cSVD MRI markers might be more important than others in the association with BBB permeability. Our results showed that Fazekas score explained similar or even higher proportions of variance in BBB permeability while dichotomized WMH explained smaller proportions of variance, probably because a wider range of severity was captured by the Fazekas score (ranging 0–6) compared with the cSVD score (ranging 0–4). However, we advocate cSVD score as an alternative to Fazekas scale, because it may provide a more comprehensive overview of cSVD-related brain damage.

Apart from BBB permeability change, we also found that participants with higher total MRI cSVD burden had a lower fractional blood plasma volume in the NAWM, CGM, and DGM. As blood plasma volume is related to the CBF, this finding is in line with a previous study (29), further illustrating the association between the cSVD and reduced CBF. A meta-analysis of 4 longitudinal and 34 cross-sectional CBF studies also showed that CBF was lower in subjects with more WMH, and most CBF data were from gray matter, which was consistent with our study (30). These results further confirmed the hypothesis that cSVD and CBF are associated with chronic hypoperfusion or impaired cerebrovascular reactivity (1). However, there was no association between higher total MRI cSVD burden and lower fractional blood plasma volume in the WMH in our study. There are three possible reasons. First, in the meta-analysis mentioned earlier, most included studies recorded CBF in CGM, data for white matter were limited, thus, this research emphasized that more CBF data are needed for white matter, especially separate data for NAWM and WMH. Second, some longitudinal studies revealed that more baseline WMH predated falling CBF, which suggested that hypoperfusion was more likely a consequence of WMH than the cause. Therefore, it is still unclear whether reduced CBF is the etiology of WMH or secondary reaction of decreased metabolism of injured WM areas. Third, another longitudinal study (31) showed that decreasing CBF was related to progression of PVWMH rather than to that of DWMH. But they also found no association between CBF and volume of total WMH, PVWMH, or DWMH at baseline. The contradictory follow-up result indicated that the vulnerability for DWMH and PVWMH might be different since these two brain areas are on different sections of the arteriolar tree. PVWMH are often located symmetrically in both cerebral hemispheres, that is suggestive of diffuse perfusion disturbance but DWMH frequently have an asymmetrical distribution that is suggestive of local perfusion disturbances. But in our study, we focused on the BBB permeability of overall white matter of the brain, and each ROI was measured for four times and averaged to obtain average parameters. Therefore, we did not divide the white matter into PVWM and DWM. Further studies are required to investigate how hypoperfusion varies across different tissues by using area stratification analysis, and how it changes across the course of the cSVD.

Our study has the following strengths: (1) we used total MRI cSVD burden to analyze the association between cSVD and BBB permeability, which was a more representative and comprehensive method to reflect the severity of cSVD; (2) we applied strict exclusion criteria to avoid the effect of BBB permeability change caused by symptomatic stroke; and (3) the Patlak pharmacokinetics we applied is the most suited method to distinguish cSVD-related from age-related BBB permeability change so far (8).

Our study is also subjected to a number of limitations: (1) all MRI markers are dichotomized in the scale, locations and numbers of lacunes, and CMBs are not accounted for in this score; total quantitative load of WMH and EPVS, greater granularity for lacunes as well as CMBs and different weightings and cut points for different cSVD features should be tested in future studies; (2) since we selected participants who received physical examination from a single center, the generalizability of our results to community population may be limited; (3) our study is incapable of investigating whether the change of BBB permeability could predict cSVD progression; longitudinal studies with community-based series of participants are needed to determine whether BBB precedes or follows the various features of cSVD; and (4) in our research, there are five participants who did not have visible WMH, their WMH ROI was placed around the periventricular, which may cause an ambiguity. But in our study, we focused on the relationship between BBB permeability and total MRI cSVD burden, participants without visible WMH may have other cSVD markers, we worried that there would be selection bias if we removed these people. And participants without visible WMH were also not taken out in other similar researches (8, 9, 32). Thus, to avoid selection bias and improve the comparability with similar studies, participants without visible WMH were not excluded from this study.

## Conclusion

This study indicated that a higher total MRI cSVD burden was associated with larger BBB permeability in the NAWM, WMH, CGM, and DGM, which provides additional evidence that compromised BBB integrity may play a role in the pathogenesis of cSVD. We also observed that participants with a higher total MRI cSVD burden had a lower fractional blood plasma volume in the NAWM, CGM, and DGM. Longitudinal studies are required to confirm a causal relationship between the BBB permeability and cSVD progression.

## Ethics Statement

All participants consented to participate in our study and signed an informed consent to the use of data for research. The design of this study was approved by the Ethics Committee of Beijing Chao-Yang Hospital, Capital Medical University and was performed in accordance with the declaration of Helsinki. The details of each participant are displayed in Tables S8 in Supplementary Material.

## Author Contributions

Conception and design of the research: TJ and WH. Acquisition of the data: YL, ML, and QS. Analysis and interpretation of the data: YL, ML, and QS. Drafting the manuscript: YL and ML. Critical revision of the manuscript: LZ, WQ, LY, TJ, and WH. Final approval of the version to be published: YL, ML, LZ, QS, WQ, LY, TJ, and WH.

## Conflict of Interest Statement

Author QS was employed by company Siemens Healthcare Ltd. All other authors declare no competing interests.
