# Supplementary material for: Compromised Blood–Brain Barrier Integrity Is Associated With Total Magnetic Resonance Imaging Burden of Cerebral Small Vessel Disease
Source: Front Neurol. 2018 Apr 6;9:221. doi: 10.3389/fneur.2018.00221 (PMC5897516; doi:10.3389/fneur.2018.00221)
Supplement: Supplementary file 1 [file Table_1.doc]

| SEQUENCE | FLAIR | T2WI | T1WI | DCE-MRI | SWI |
| --- | --- | --- | --- | --- | --- |
| ORIENTATION | AX | AX | AX | AX | AX |
| TR | 9000ms | 5000ms | 2000ms | 5.08ms | 28ms |
| TE | 81ms | 94ms | 9ms | 1.8ms | 20ms |
| FOV | 24 | 24 | 24 | 24 | 24 |
| SLICE THICK. | 5mm | 5mm | 5mm | 3mm | 1.2mm |
| SLICE GAP | 6.5mm | 6.5mm | 6.5mm | 0mm | 0mm |
| MATRIX | 320×256 | 320×320 | 320×217 | 192×154 | 384×269 |
| FLIP ANGLE |  |  |  | 15° |  |
| DCE-INTERVAL |  |  |  | 3.8sec |  |

**Supplementary Table 1 Scanning parameters**
