# Supplementary material for: Compromised Blood–Brain Barrier Integrity Is Associated With Total Magnetic Resonance Imaging Burden of Cerebral Small Vessel Disease
Source: Front Neurol. 2018 Apr 6;9:221. doi: 10.3389/fneur.2018.00221 (PMC5897516; doi:10.3389/fneur.2018.00221)
Supplement: Supplementary file 2 [file Table_2.docx]

**Supplementary Table 2** **Laboratory tests of participants with different severity of total MRI cSVD burden**

| SVD Score | Total  (n = 99) | cSVD 0  (n = 31) | cSVD 1  (n = 25) | cSVD 2  (n = 16) | cSVD 3  (n = 15) | cSVD 4  (n = 12) | *P* |
| --- | --- | --- | --- | --- | --- | --- | --- |
| TC, mmol/L | 4.23 ± 0.83 | 4.35 ± 0.95 | 4.22 ± 0.87 | 4.34 ± 0.80 | 3.92 ± 0.61 | 4.16 ± 0.70 | 0.541 |
| TG, mmol/L | 1.28 (0.92, 1.60) | 1.06 (0.91, 1.33) | 1.30 (0.86, 2.04) | 1.49 (0.88, 2.07) | 1.32 (1.06, 1.52) | 1.45 (1.28, 1.68) | 0.210 |
| HDL, mmol/L | 1.10 (0.90, 1.30) | 1.10 (0.90, 1.30) | 1.00 (0.90, 1.20) | 1.25 (1.00, 1.48) | 1.10 (0.90, 1.50) | 1.20 (0.93, 1.30) | 0.696 |
| LDL, mmol/L | 2.30 (1.90, 3.20) | 2.90 (2.10, 3.30) | 2.70 (1.80, 3.25) | 2.20 (1.90, 2.58) | 2.20 (1.90, 2.50) | 2.30 (1.83, 3.28) | 0.445 |
| HbA1c, % | 5.70 (5.40, 6.00) | 5.50 (5.40, 5.80) | 5.80 (5.55, 5.95) | 5.80 (5.43, 6.83) | 5.50 (5.40, 5.90) | 6.00 (5.73, 6.60) | 0.058 |
| HCY, mmol/L | 14.00 (12.00, 17.00) | 13.00 (12.00, 16.00) | 13.00 (11.50, 15.00) | 16.50 (13.25, 23.00) | 15.00 (12.00, 17.00) | 15.00 (12.25, 17.00) | 0.142 |
| hs-CRP, mg/L | 1.30 (0.71, 2.16) | 1.54 (0.58, 2.13) | 1.35 (0.74, 3.01) | 1.15 (0.78, 2.12) | 1.30 (0.98, 2.03) | 0.94 (0.41, 2.96) | 0.755 |
| Uric, μmol/L | 321.47 ± 94.10 | 312.39 ± 100.10 | 341.84 ± 74.96 | 330.56 ± 112.82 | 297.73 ± 89.42 | 320.08 ± 98.45 | 0.641 |
| Cr, μmol/L | 65.00 (54.80, 76.20) | 61.50 (51.80, 81.30) | 72.40 (53.85, 80.10) | 63.55 (56.60, 67.38) | 63.60 (58.00, 73.80) | 72.60 (56.08, 87.80) | 0.785 |

Data are presented as mean± standard deviation or median (interquartile range).

MRI indicates magnetic resonance imaging; cSVD, cerebral small vessel disease; TC, total cholesterol; TG, triglyceride; HDL, high density lipoprotein; LDL, low density lipoprotein; HbA1c, glycosylated hemoglobin; HCY, homocysteine; hs-CRP, high-sensitivity C-reactive protein; Uric, uric acid; and Cr, serum creatinine.
