# Supplementary material for: Compromised Blood–Brain Barrier Integrity Is Associated With Total Magnetic Resonance Imaging Burden of Cerebral Small Vessel Disease
Source: Front Neurol. 2018 Apr 6;9:221. doi: 10.3389/fneur.2018.00221 (PMC5897516; doi:10.3389/fneur.2018.00221)
Supplement: Supplementary file 3 [file Table_3.docx]

**Supplementary Table 3 Demographic and clinical features of participants** **with different severity of total MRI cSVD burden**

|  | Total  (n = 94) | cSVD 0  (n = 27) | cSVD 1  (n = 24) | cSVD 2  (n = 16) | cSVD 3  (n = 15) | cSVD 4  (n = 12) | *P* |
| --- | --- | --- | --- | --- | --- | --- | --- |
| Male, n(%) | 46 (48.9) | 11 (40.7) | 14 (58.3) | 5 (31.3) | 8 (53.3) | 8 (66.7) | 0.267 |
| Age, years | 70.61 ± 9.10 | 68.00 ± 9.22 | 70.38 ± 8.04 | 71.06 ± 7.86 | 75.47 ± 10.22 | 70.25 ± 9.87 | 0.159 |
| Hypertension, n(%) | 64 (68.1) | 16 (59.3) | 16 (66.7) | 11 (68.8) | 13 (86.7) | 8 (66.7) | 0.495 |
| Diabetes mellitus, n(%) | 19 (20.2) | 5 (18.5) | 2 (8.3) | 4 (25.0) | 3 (20.0) | 5 (41.7) | 0.215 |
| Hyperlipidemia, n(%) | 51 (54.3) | 13 (48.1) | 14 (58.3) | 9 (56.3) | 9 (60.0) | 6 (50.0) | 0.928 |
| Current smoking, n(%) | 27 (28.7) | 6 (22.2) | 9 (37.5) | 5 (31.3) | 3 (20.0) | 4 (33.3) | 0.700 |
| BMI, kg/m^2^ | 25.84 ± 3.18 | 25.63 ± 2.50 | 26.40 ± 4.26 | 26.07 ± 2.66 | 25.37 ± 3.40 | 25.44 ± 2.66 | 0.841 |

Data are presented as mean ± standard deviation, median (interquartile range) or counts (%).

MRI indicates magnetic resonance imaging; cSVD, cerebral small vessel disease; n, number of persons; and BMI, body mass index.
