# Supplementary material for: Compromised Blood–Brain Barrier Integrity Is Associated With Total Magnetic Resonance Imaging Burden of Cerebral Small Vessel Disease
Source: Front Neurol. 2018 Apr 6;9:221. doi: 10.3389/fneur.2018.00221 (PMC5897516; doi:10.3389/fneur.2018.00221)
Supplement: Supplementary file 4 [file Table_4.docx]

**Supplementary Table 4 Leakage rate, area under the leakage curve and fractional blood plasma volume of participants**

**with different severity of total MRI cSVD burden**

|  | cSVD 0  (n = 27) | cSVD 1  (n = 24) | cSVD 2  (n = 16) | cSVD 3  (n = 15) | cSVD 4  (n = 12) | *P* |
| --- | --- | --- | --- | --- | --- | --- |
| NAWM |  |  |  |  |  |  |
| K_trans_ (10^-4^min^-1^) | 0.14 (0.03, 0.34) ^b,c^ | 0.18 (0.10, 0.37) ^f^ | 0.30 (0.22, 0.43) | 0.37 (0.22, 0.48) ^b^ | 0.47 (0.35, 0.55) ^c,f^ | ＜0.001 |
| AUC | 3.31 ± 1.07 ^a,b,c^ | 3.63 ± 0.96 ^d,e,f^ | 4.75 ± 0.64 ^a,d^ | 4.45 ± 1.43 ^b,e^ | 4.93 ± 1.43 ^c,f^ | ＜0.001 |
| V_p_ (10^-2^) | 6.49 ± 2.50 ^a,b^ | 5.88 ± 2.44 | 4.76 ± 2.21 ^a^ | 4.73 ± 2.00 ^b^ | 5.29 ± 1.90 | 0.045 |
| WMH |  |  |  |  |  |  |
| K_trans_ (10^-4^min^-1^) | 0.28 (0.11, 0.43) ^a,b,c^ | 0.38 (0.19, 0.55) ^f^ | 0.57 (0.33, 0.94) ^a^ | 0.56 (0.39, 0.87) ^b^ | 0.67 (0.55, 0.93) ^c,f^ | ＜0.001 |
| AUC | 4.00 ± 1.25 ^a,b,c^ | 4.50 ± 1.27 ^d,e,^^f^ | 6.20 ± 1.89 ^a,d^ | 6.50 ± 1.79 ^b,e^ | 7.05 ± 1.82 ^c,f^ | ＜0.001 |
| V_p_ (10^-2^) | 9.36 ± 5.34 | 8.27 ± 4.27 | 9.37 ± 5.15 | 8.43 ± 5.28 | 6.62 ± 3.10 | 0.517 |
| CGM |  |  |  |  |  |  |
| K_trans_ (10^-4^min^-1^) | 0.93 (0.70, 1.26) ^b,c^ | 0.98 (0.78, 1.32) ^e^ | 1.36 (0.99, 2.39) | 1.91 (1.12, 2.44) ^b,e^ | 1.59 (1.21, 2.03) ^c^ | ＜0.001 |
| AUC | 14.58 ± 5.62 ^a,b,c^ | 15.59 ± 4.99 ^d,f^ | 19.22 ± 4.81 ^a,d^ | 18.55 ± 4.98 ^b^ | 20.02 ± 5.29 ^c,f^ | 0.005 |
| V_p_ (10^-2^) | 27.31 ± 10.11 ^c^ | 24.45 ± 9.40 ^f^ | 23.42 ± 9.84 | 22.20 ± 10.05 | 17.91 ± 7.04 ^c,f^ | 0.046 |
| DGM |  |  |  |  |  |  |
| K_trans_ (10^-4^min^-1^) | 0.56 (0.35, 0.74) ^b,c^ | 0.62 (0.49, 0.90) ^f^ | 0.72 (0.58, 1.05) | 0.96 (0.67, 1.53) ^b^ | 1.00 (0.91, 1.47) ^c,f^ | ＜0.001 |
| AUC | 9.74 ± 3.06 ^a,c^ | 10.53 ± 2.31 ^f^ | 11.92 ± 2.15 ^a^ | 11.17 ± 2.88 | 12.37 ± 2.06 ^c,f^ | 0.019 |
| V_p_ (10^-2^) | 18.48 ± 6.85 ^b,c^ | 17.91 ± 7.99 ^f^ | 14.75 ± 5.86 | 14.14 ± 6.19 ^b^ | 11.56 ± 5.44 ^c,f^ | 0.019 |

Data are presented as mean ± standard deviation or median (interquartile range).

MRI indicates magnetic resonance imaging; cSVD, cerebral small vessel disease; NAWM, normal-appearing white matter; WMH, white matter

hyperintensities; CGM, cortex gray matter; DGM, deep gray matter; K_trans_, leakage rate; AUC, area under the leakage curve; and V_p_, fractional blood

plasma volume.

a. Significant difference between cSVD 0 and cSVD 2 categories;

b. Significant difference between cSVD 0 and cSVD 3 categories;

c. Significant difference between cSVD 0 and cSVD 4 categories;

d. Significant difference between cSVD 1 and cSVD 2 categories;

e. Significant difference between cSVD 1 and cSVD 3 categories;

f. Significant difference between cSVD 1 and cSVD 4 categories.
