# Supplementary material for: Compromised Blood–Brain Barrier Integrity Is Associated With Total Magnetic Resonance Imaging Burden of Cerebral Small Vessel Disease
Source: Front Neurol. 2018 Apr 6;9:221. doi: 10.3389/fneur.2018.00221 (PMC5897516; doi:10.3389/fneur.2018.00221)
Supplement: Supplementary file 5 [file Table_5.docx]

**Supplementary Table 5 Association of leakage rate, area under the leakage curve and fractional blood plasma volume with** **total MRI cSVD burden**

|  | Spearman correlation | | Univariable^a^ | | Multivariable^b^ | |
| --- | --- | --- | --- | --- | --- | --- |
|  | r | *P* Value | β | *P* Value | β | *P* Value |
| NAWM |  |  |  |  |  |  |
| K_trans_ | 0.550 | < 0.001 | 0.081 | < 0.001 | 0.081 | < 0.001 |
| AUC | 0.455 | < 0.001 | 0.425 | < 0.001 | 0.425 | < 0.001 |
| V_p_ | -0.218 | 0.035 | -0.419 | 0.017 | -0.419 | 0.017 |
| WMH |  |  |  |  |  |  |
| K_trans_ | 0.561 | < 0.001 | 0.147 | < 0.001 | 0.150 | < 0.001 |
| AUC | 0.594 | < 0.001 | 0.832 | < 0.001 | 0.863 | < 0.001 |
| V_p_ | -0.107 | 0.304 | -0.468 | 0.192 | -0.352 | 0.345 |
| CGM |  |  |  |  |  |  |
| K_trans_ | 0.496 | < 0.001 | 0.241 | < 0.001 | 0.225 | < 0.001 |
| AUC | 0.393 | < 0.001 | 1.447 | 0.001 | 1.685 | < 0.001 |
| V_p_ | -0.261 | 0.011 | -2.050 | 0.004 | -1.867 | 0.008 |
| DGM |  |  |  |  |  |  |
| K_trans_ | 0.486 | < 0.001 | 0.169 | < 0.001 | 0.169 | < 0.001 |
| AUC | 0.353 | < 0.001 | 0.616 | 0.002 | 0.616 | 0.002 |
| V_p_ | -0.319 | 0.002 | -1.731 | 0.001 | -1.604 | 0.001 |

MRI indicates magnetic resonance imaging; cSVD, cerebral small vessel disease; NAWM, normal-appearing white matter; WMH, white matter hyperintensities; CGM, cortex gray matter; DGM, deep gray matter; K_trans_, leakage rate; AUC, area under the leakage curve; and V_p_, fractional blood plasma volume.

a: Univariable linear regression analysis with K_trans_, AUC, and V_p_, respectively, as dependent variable, and total MRI cSVD burden as independent variable.

b: Multivariable linear regression analysis with K_trans_, AUC, and V_p_ as dependent variable and age, sex, total MRI cSVD burden, and vascular risk factors as independent variables.

β: Unstandardized regression coefficient.
