# Supplementary material for: Compromised Blood–Brain Barrier Integrity Is Associated With Total Magnetic Resonance Imaging Burden of Cerebral Small Vessel Disease
Source: Front Neurol. 2018 Apr 6;9:221. doi: 10.3389/fneur.2018.00221 (PMC5897516; doi:10.3389/fneur.2018.00221)
Supplement: Supplementary file 6 [file Table_6.docx]

**Supplementary Table 6 Association of leakage rate, area under the leakage curve and fractional blood plasma volume with** **total MRI cSVD burden (without WMH)**

|  | Spearman correlation | | Univariable^a^ | | Multivariable^b^ | |
| --- | --- | --- | --- | --- | --- | --- |
|  | r | *P* Value | β | *P* Value | β | *P* Value |
| NAWM |  |  |  |  |  |  |
| K_trans_ | 0.518 | < 0.001 | 0.104 | < 0.001 | 0.104 | < 0.001 |
| AUC | 0.350 | < 0.001 | 0.477 | < 0.001 | 0.477 | < 0.001 |
| V_p_ | -0.263 | 0.008 | -0.655 | 0.005 | -0.655 | 0.005 |
| WMH |  |  |  |  |  |  |
| K_trans_ | 0.535 | < 0.001 | 0.187 | < 0.001 | 0.180 | < 0.001 |
| AUC | 0.481 | < 0.001 | 0.940 | < 0.001 | 1.030 | < 0.001 |
| V_p_ | -0.156 | 0.124 | -0.808 | 0.081 | -0.556 | 0.260 |
| CGM |  |  |  |  |  |  |
| K_trans_ | 0.429 | < 0.001 | 0.309 | < 0.001 | 0.289 | < 0.001 |
| AUC | 0.306 | 0.002 | 1.489 | 0.005 | 1.712 | 0.001 |
| V_p_ | -0.295 | 0.003 | -2.895 | 0.002 | -2.895 | 0.002 |
| DGM |  |  |  |  |  |  |
| K_trans_ | 0.440 | < 0.001 | 0.216 | < 0.001 | 0.216 | < 0.001 |
| AUC | 0.307 | 0.002 | 0.783 | 0.003 | 0.690 | 0.009 |
| V_p_ | -0.315 | 0.002 | -2.193 | 0.001 | -2.193 | 0.001 |

MRI indicates magnetic resonance imaging; cSVD, cerebral small vessel disease; NAWM, normal-appearing white matter; WMH, white matter hyperintensities; CGM, cortex gray matter; DGM, deep gray matter; K_trans_, leakage rate; AUC, area under the leakage curve; and V_p_, fractional blood plasma volume.

a: Univariable linear regression analysis with K_trans_, AUC, and V_p_, respectively, as dependent variable, and total MRI cSVD burden (without WMH) as independent variable.

b: Multivariable linear regression analysis with K_trans_, AUC, and V_p_ as dependent variable and age, sex, total MRI cSVD burden (without WMH), and vascular risk factors as independent variables.

β: Unstandardized regression coefficient.
