# Supplementary material for: Compromised Blood–Brain Barrier Integrity Is Associated With Total Magnetic Resonance Imaging Burden of Cerebral Small Vessel Disease
Source: Front Neurol. 2018 Apr 6;9:221. doi: 10.3389/fneur.2018.00221 (PMC5897516; doi:10.3389/fneur.2018.00221)
Supplement: Supplementary file 7 [file Table_7.docx]

**Supplementary Table 7 R^2^ for the association between** **BBB** **permeability and total MRI cSVD burden versus individual MRI markers**

|  | Total MRI cSVD burden | | Total MRI cSVD burden  (without WMH) | | Lacunes | | WMH | | CMBs | | EPVS | |
| --- | --- | --- | --- | --- | --- | --- | --- | --- | --- | --- | --- | --- |
|  | R^2^ | *P* | R^2^ | *P* | R^2^ | *P* | R^2^ | *P* | R^2^ | *P* | R^2^ | *P* |
| NAWM |  |  |  |  |  |  |  |  |  |  |  |  |
| K_trans_ | 0.220 | < 0.001 | 0.202 | < 0.001 | 0.150 | < 0.001 | 0.127 | < 0.001 | 0.106 | 0.001 | 0.082 | 0.004 |
| AUC | 0.170 | < 0.001 | 0.152 | < 0.001 | 0.054 | 0.021 | 0.194 | < 0.001 | 0.133 | < 0.001 | 0.049 | 0.028 |
| V_p_ | 0.087 | 0.003 | 0.078 | 0.005 | 0.058 | 0.017 | 0.054 | 0.021 | 0.008 | 0.392 | 0.077 | 0.005 |
| WMH |  |  |  |  |  |  |  |  |  |  |  |  |
| K_trans_ | 0.265 | < 0.001 | 0.237 | < 0.001 | 0.079 | 0.005 | 0.164 | < 0.001 | 0.113 | 0.001 | 0.214 | < 0.001 |
| AUC | 0.357 | < 0.001 | 0.256 | < 0.001 | 0.114 | 0.001 | 0.353 | < 0.001 | 0.207 | < 0.001 | 0.118 | 0.001 |
| V_p_ | 0.023 | 0.132 | 0.031 | 0.081 | 0.042 | 0.041 | 0.003 | 0.601 | 0.005 | 0.479 | 0.012 | 0.288 |
| CGM |  |  |  |  |  |  |  |  |  |  |  |  |
| K_trans_ | 0.181 | < 0.001 | 0.165 | < 0.001 | 0.141 | < 0.001 | 0.108 | 0.001 | 0.036 | 0.060 | 0.105 | 0.001 |
| AUC | 0.117 | 0.001 | 0.078 | 0.005 | 0.018 | 0.190 | 0.123 | < 0.001 | 0.054 | 0.020 | 0.067 | 0.010 |
| V_p_ | 0.094 | 0.002 | 0.097 | 0.002 | 0.092 | 0.002 | 0.039 | 0.051 | 0.037 | 0.055 | 0.037 | 0.058 |
| DGM |  |  |  |  |  |  |  |  |  |  |  |  |
| K_trans_ | 0.233 | < 0.001 | 0.208 | < 0.001 | 0.128 | < 0.001 | 0.146 | < 0.001 | 0.115 | 0.001 | 0.101 | 0.001 |
| AUC | 0.110 | 0.001 | 0.085 | 0.003 | 0.075 | 0.006 | 0.094 | 0.002 | 0.034 | 0.066 | 0.035 | 0.064 |
| V_p_ | 0.117 | 0.001 | 0.109 | 0.001 | 0.084 | 0.004 | 0.034 | 0.069 | 0.011 | 0.291 | 0.039 | 0.051 |

R^2^ indicates Coefficient of determination; cSVD, cerebral small vessel disease; WMH, white matter hyperintensities; CMBs, cerebral microbleeds; EPVS, enlarged perivascular spaces; NAWM, normal-appearing white matter; CGM, cortex gray matter; DGM, deep gray matter; K_trans_, leakage rate; AUC, area under the leakage curve; and V_p_, fractional blood plasma volume.
