# Supplementary material for: Compromised Blood–Brain Barrier Integrity Is Associated With Total Magnetic Resonance Imaging Burden of Cerebral Small Vessel Disease
Source: Front Neurol. 2018 Apr 6;9:221. doi: 10.3389/fneur.2018.00221 (PMC5897516; doi:10.3389/fneur.2018.00221)
Supplement: Supplementary file 8 [file Table_8.docx]

Supplementary Table 8 The reasons for the participants to choose physical examination

| Number | Hypertension | Diabetes mellitus | Hyperlipidemia | Family history of cerebrovascular disease |
| --- | --- | --- | --- | --- |
| 1 | YES | YES | YES | NO |
| 2 | NO | NO | NO | YES |
| 3 | NO | NO | NO | YES |
| 4 | YES | NO | YES | NO |
| 5 | YES | YES | YES | NO |
| 6 | NO | NO | NO | YES |
| 7 | YES | YES | YES | NO |
| 8 | YES | YES | YES | YES |
| 9 | NO | NO | NO | YES |
| 10 | YES | YES | YES | NO |
| 11 | YES | NO | NO | YES |
| 12 | YES | NO | YES | NO |
| 13 | NO | YES | YES | NO |
| 14 | YES | NO | NO | NO |
| 15 | YES | NO | YES | NO |
| 16 | NO | YES | NO | YES |
| 17 | YES | NO | NO | NO |
| 18 | YES | NO | YES | NO |
| 19 | YES | NO | NO | NO |
| 20 | NO | YES | NO | YES |
| 21 | YES | NO | YES | NO |
| 22 | YES | NO | YES | NO |
| 23 | YES | NO | YES | NO |
| 24 | NO | NO | NO | YES |
| 25 | NO | NO | YES | NO |
| 26 | YES | NO | NO | NO |
| 27 | YES | NO | YES | NO |
| 28 | YES | YES | YES | YES |
| 29 | YES | NO | NO | NO |
| 30 | NO | NO | NO | YES |
| 31 | YES | YES | YES | NO |
| 32 | YES | NO | NO | NO |
| 33 | YES | NO | YES | NO |
| 34 | YES | NO | NO | NO |
| 35 | NO | NO | YES | NO |
| 36 | NO | YES | NO | NO |
| 37 | NO | YES | NO | YES |
| 38 | YES | YES | NO | NO |
| 39 | YES | NO | NO | NO |
| 40 | NO | YES | YES | NO |
| 41 | NO | NO | YES | NO |
| 42 | YES | NO | NO | NO |
| 43 | YES | YES | YES | NO |
| 44 | YES | NO | NO | NO |
| 45 | YES | NO | YES | NO |
| 46 | NO | NO | NO | YES |
| 47 | NO | NO | NO | YES |
| 48 | YES | NO | YES | NO |
| 49 | YES | YES | YES | NO |
| 50 | YES | NO | YES | NO |
| 51 | YES | NO | NO | NO |
| 52 | YES | NO | NO | NO |
| 53 | YES | NO | YES | NO |
| 54 | YES | NO | YES | NO |
| 55 | YES | NO | YES | NO |
| 56 | NO | NO | NO | YES |
| 57 | YES | NO | YES | YES |
| 58 | NO | NO | YES | NO |
| 59 | YES | NO | YES | NO |
| 60 | YES | NO | YES | YES |
| 61 | YES | NO | YES | NO |
| 62 | YES | NO | YES | NO |
| 63 | NO | NO | YES | NO |
| 64 | NO | NO | NO | YES |
| 65 | NO | NO | YES | NO |
| 66 | NO | NO | NO | YES |
| 67 | NO | NO | NO | YES |
| 68 | YES | NO | NO | NO |
| 69 | NO | YES | NO | NO |
| 70 | YES | NO | YES | NO |
| 71 | NO | NO | NO | YES |
| 72 | YES | NO | YES | NO |
| 73 | YES | NO | YES | YES |
| 74 | YES | NO | YES | YES |
| 75 | NO | YES | YES | NO |
| 76 | YES | NO | YES | NO |
| 77 | NO | NO | NO | YES |
| 78 | YES | NO | YES | YES |
| 79 | YES | NO | YES | NO |
| 80 | YES | NO | NO | NO |
| 81 | YES | NO | YES | NO |
| 82 | YES | NO | NO | NO |
| 83 | NO | NO | YES | NO |
| 84 | YES | NO | NO | NO |
| 85 | NO | NO | NO | YES |
| 86 | NO | NO | YES | NO |
| 87 | YES | YES | NO | YES |
| 88 | NO | NO | NO | YES |
| 89 | NO | NO | YES | NO |
| 90 | YES | NO | NO | NO |
| 91 | YES | NO | NO | NO |
| 92 | YES | NO | NO | NO |
| 93 | YES | NO | NO | YES |
| 94 | YES | NO | YES | NO |
| 95 | YES | NO | YES | NO |
| 96 | YES | NO | YES | YES |
| 97 | YES | NO | NO | NO |
| 98 | NO | NO | NO | YES |
| 99 | YES | NO | YES | NO |
